# Composite Aerogel Comprised of Sodium Alginate and Bentonite via Supercritical CO_2_ Drying: An Efficient Adsorbent for Lysozyme

**DOI:** 10.3390/gels8060359

**Published:** 2022-06-08

**Authors:** Jie Zhao, Liqin Cao, Yong Dong

**Affiliations:** 1 Key Laboratory of Oil and Gas Fine Chemicals, Ministry of Education & Xinjiang Uygur Autonomous Region, Xinjiang University, Urumqi 830017, China; cao_lq@xju.edu.cn (J.Z.); xj_yongdong@163.com (Y.D.); 2 State Key Laboratory of Chemistry and Utilization of Carbon Based Energy Resources, College of Chemistry, Xinjiang University, Urumqi 830017, China

**Keywords:** aerogels, composites, sodium alginate, bentonite, supercritical CO_2_-drying

## Abstract

To meet the demand for the separation of specific substances, the construction of porous composite aerogels with a high specific surface area and a strong adsorption capacity is still a challenge. Herein, a sodium alginate/bentonite composite aerogel was efficiently prepared through supercritical fluid drying. The aerogel’s volume shrank less during supercritical drying, maintaining its original three-dimensional mesh structure. The resulting aerogel had a large specific surface area (445 m^2^/g), a low density (0.059 g/cm^3^), and a large pore volume (3.617 cm^3^/g). Due to the fixation and intercalation effects, bentonite was uniformly dispersed in the sodium alginate matrixes. The adsorption of lysozyme by the composite aerogel was evaluated, and the results showed that the optimal adsorption pH was 8 when the pH of the phosphoric acid buffer solution was between pH = 5 and 8.5. The time for adsorption to reach equilibrium was 8 h. The adsorption capacity increased with the increase in bentonite content, and when the initial concentration of lysozyme was from 0.2 to 1.2 g/L, the adsorption capacity first increased and then stabilized, and the maximum adsorption amount was 697 mg/g. The adsorption behavior was simulated in the isothermal region, and the linear correlation coefficient of Langmuir isothermal adsorption fitting was found to be 0.997. Thus, this composite aerogel with strong adsorption capacity can be used as a good alternative to enzymatic adsorbents or immobilized materials.

## 1. Introduction

Functional porous materials are widely used for enzyme immobilization and protein isolation/purification. This may be due to their potential advantages for material microstructures. Among them, the construction of hydrogel and the thereby-derived aerogel for protein or biological polypeptide adsorption after the drying process has aroused increasing research interest, mainly owing to its good application performance, feasible engineering operation, and environmentally friendly properties [1]. An aerogel is a lightweight solid material with a high porosity, low density, and high specific surface area. The types of aerogels include silicon, carbon, sulfur, metal oxide, etc. The interior contains a large amount of air, which can play a role in heat insulation, and the low density is due to it being an ultra-light material with a nano-sized pore structure, so it has broad application prospects in aerospace, energy storage, adsorption, catalysis, thermal insulation, and other fields [2,3,4]. Quignard and colleagues recently reviewed aerogel materials from marine polysaccharides, of which alginates with a high guluronic content gave gels with a higher strength due to the stronger affinity of the guluronic residues for divalent cations [5]. In addition, the common methods of drying the hydrocolloid to create the aerogel are freeze drying and supercritical CO_2_ (scCO_2_) drying. Supercritical drying yields aerogels that are endowed with higher porosities and higher surface areas when compared to freeze drying and ambient pressure drying [6]. In our previous study, we developed a poly (4-vinylpyridine) crosslinked SiO_2_ (P4VP/SiO_2_) composite aerogel obtained through supercritical CO_2_ drying, which had a specific surface area of 314 m^2^/g and was endowed with an enhanced adsorption capacity for copper ions [7].

Alginate (Alg), a type of anionic natural polysaccharide with good biocompatibility and biodegradability, is a pH-sensitive raw material that is extensively obtained from brown algae [8,9]. Due to its abundance and relatively low price, alginate has been extensively applied in the biomaterial manufacturing, the food industry, and the biomedical field for tissue engineering, controlled drug release, etc. Based on the pH-sensitive properties of alginate, García-González et al. reported an alginate aerogel loaded with ketoprofen as a pH-responsive drug carrier with a faster drug release rate [10]. Lu and colleagues reported a novel dual-responsive Alg-based aerogel with thermo-/pH-sensitive properties that was applied as controlled drug release system [11]. Amaly and colleagues reported sponge alginate beads that exhibited favorable protein adsorption performance, along with reusability and selectivity [12].

The main component of bentonite (Bt) is montmorillonite, whose content is 80–90%. Montmorillonite is composed of a silicon–oxygen tetrahedron sandwiched with an aluminum–oxygen octahedron, and there are cations such as Cu^2+^, Mg^2+^, Ca^2+^, Na^+^, K^+^, and others between the layered structures [13,14]. Bt can expand 20–30 times after absorbing water. Because of its good physical and chemical properties, it can be used as a purification decolorizer, binder, thixotropic agent, suspension agent, stabilizer, filler, feed, catalyst, etc., and it is widely used in agriculture, light industry, cosmetics, pharmaceuticals, and other fields, so it is called “universal clay” [15]. In particular, Shamsuddin reported a protein-intercalated bentonite for a bioplastic nanocomposite [16]. Because Bt has divalent metal ions, sodium alginate can exhibit an ion-exchange reaction and can physically crosslink through it [17]. Thus, the ions can be firmly connected and can form a mechanically stable nanocomposite. Some researchers have reported the immobilization of horseradish peroxidase, pectinase, and *Candida rugosa* lipase on Alg-/clay-based hydrogels for biotechnological applications [18,19,20]. Lysozyme is one of the most abundant antimicrobial proteins. However, a study on incorporating lysozyme into Alg-/clay-composite hydrogels has not been reported.

To the best of our knowledge, a composite aerogel directly obtained via Alg-intercalated pristine Bt and supercritical CO_2_ drying has rarely been reported. Thus, the related methodology and the evaluation of its protein adsorption properties (with lysozyme as a model) should be explored. Herein, using sodium alginate and Bt as raw materials, sodium alginate/bentonite composite aerogels were prepared with sol-gel and the supercritical drying method, and the influences of the solution concentration of sodium alginate and the Bt proportion on the composite aerogel structure were determined. In addition, with lysozyme as a model protein, the adsorption performance of lysozyme on the composite aerogel was studied, and the effects of pH, adsorption time, and lysozyme concentration on the adsorption were further evaluated. Meanwhile, the adsorption behavior was simulated using the Langmuir and Freundlich isothermal adsorption models. We highlight the strong protein affinity of the composite aerogel that was directly obtained from raw materials and supercritical CO_2_ drying.

## 2. Results and Discussion

### 2.1. Characterization

**Figure 1:**
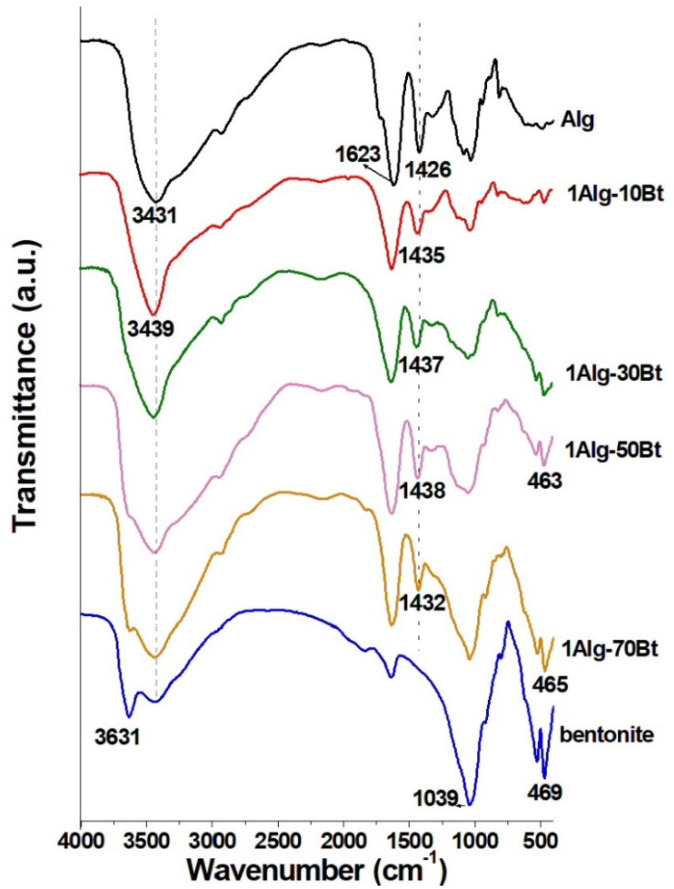
FTIR spectra of the alginate–bentonite composite aerogel.

The infrared spectra of sodium alginate, bentonite, and the composite aerogel were tested, and the results are shown in Figure 1. In the infrared spectrum of bentonite, 3631 cm^−1^ is the vibration peak of hydroxyl groups in Al-OH, 3435 cm^−1^ is the hydroxyl stretching vibration peak of Si-OH, and 1039, 799, 526, and 469 cm^−1^ are the characteristic absorption peaks of Si-O. On increasing the amount of bentonite content in the composite aerogel, the peak of Si-OH shifted toward lower frequencies, suggesting an interaction between Bt and Alg.

In the infrared spectrum of sodium alginate, 3431 cm^−1^ is the hydroxyl stretching vibration peak, 1623 and 1426 cm^−1^ are the asymmetrical and symmetrical stretching vibration peaks of COO–, 1087 cm^−1^ is the vibration peak of C–O–C, and 1032 cm^−1^ is the C–O stretching vibration peak of C–O on the pyranosyl ring. Obviously, the characteristic peaks of bentonite and sodium alginate appeared in the spectra of the composite aerogel, and with the increase in the bentonite content in the composites, the peak strength of the characteristic absorption peak at 1039 cm^−1^ of bentonite also increased. In addition, it can be seen from the spectra that the peak shape of the composite aerogel was similar to that of sodium alginate when the bentonite content was low.

**Figure 2:**
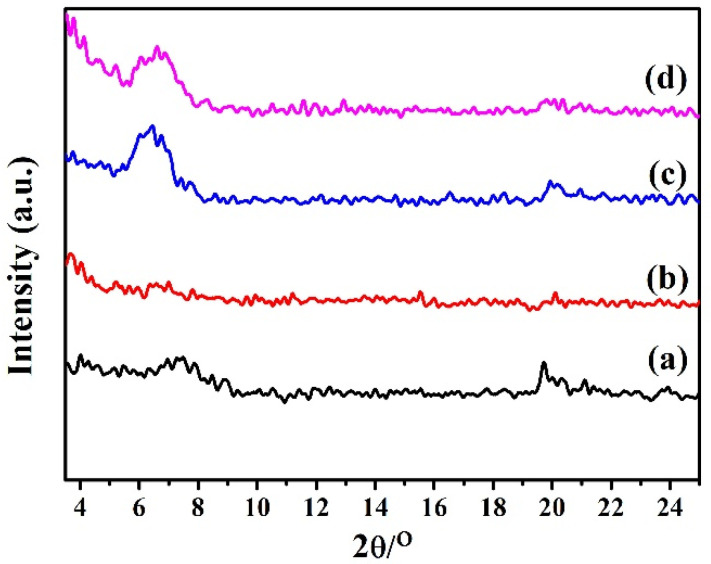
XRD curves of (**a**) bentonite, (**b**) 1Alg-10Bt, (**c**) 1Alg-30Bt, and (**d**) 3Alg-30Bt.

To investigate the interaction between sodium alginate and bentonite, the composite aerogels were tested through XRD. The results are shown in Figure 2. It can be seen that the typical diffraction peaks of bentonite in the original bentonite, 1Alg-10Bt, 1Alg-30Bt, and 3Alg-30Bt were 7.4°, 6.64°, 6.26°, and 6.6°, respectively. Through Bragg’s law, *d* = *nλ*/2sinθ, their interlayer distances (*d*-spacings) after compositing were determined to be 1.19, 1.30, 1.41, and 1.33, respectively [21,22]. From the change trends of the interlayer distances and the X-ray peak shifts of the nanocomposite aerogel, it was indicated that the sodium alginate chain was intercalated in the gallery of bentonite, resulting in an expanded gallery.

### 2.2. BET Analysis

**Figure 3:**
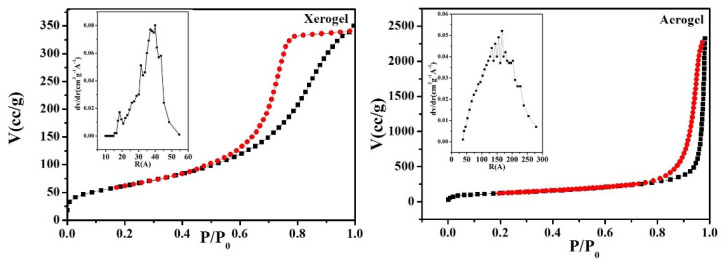
N_2_ adsorption–desorption isotherms and pore size distribution of the composite aerogel and xerogel; black square represent adsorption, red dot represent desorption.

To compare the structure and the pore size of the obtained aerogels and dry gel, N_2_ adsorption–desorption experiments were carried out via the BET method. Figure 3 shows the N_2_ adsorption–desorption isotherm curves and the results of the pore size distribution. The nitrogen BET adsorption–desorption method is often used to investigate the pores in the mesoporous (2–50 nm) range of the material. This part of the pores is only a small fraction of the pores for aerogels, but it can indicate the pore information of the connecting parts of the fibrous structures of aerogels. The hysteresis loop of dry gel (xerogel) in adsorption–desorption isotherm curves was of the H2 type, and the hysteresis loop formation may have been caused by pores with a uniform accumulation of particles, which was consistent with the result of SEM. The hysteresis loop of the aerogel in the adsorption–desorption curves could be classified as a type IV isotherm, indicating a characteristic of the mesoporous structure of the composite aerogel [5]. Meanwhile, the N_2_ adsorption capacity of the composite aerogel was significantly enhanced compared to that of the xerogel, suggesting that it was a uniform pore model, and the distribution of the mesoporous structure in the aerogel was relatively uniform.

The specific surface area, pore volume, pore size, and density of the aerogel and dry gel were tested, and the results are listed in Table 1. The results indicate that the specific surface area of the aerogel was two times higher than that of the dry gel, reaching 445 m^2^/g. The pore volume and pore diameter of the obtained aerogels were 6.6 and 3.5 times higher than those of the dry gel, with values of 3.62 cm^3^/g and 32.62 nm, respectively. Notably, the change in its density was 17 times lower than that of xerogel, and the bulk density was about 0.0596 g/cm^3^, which belongs to the typical aerogel density range.

**Figure 4:**
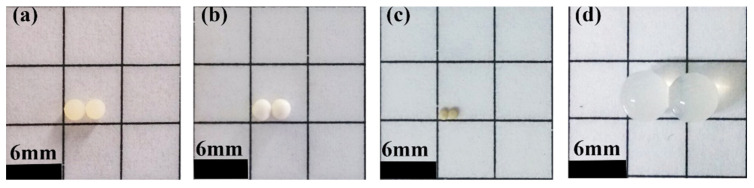
The pictures of the composite alginate gel: (**a**) alcogel, (**b**) aerogel, (**c**) xerogel, and (**d**) swelling state of the aerogel.

To confirm the advantages of scCO_2_ drying again, Figure 4 presents pictures of the gel beads in different states of existence. As shown in Figure 4, after scCO_2_ drying, the appearance of the gel was changed from translucent to white and opaque, with less volume shrinkage. The dry gel (xerogel) obtained through ambient drying underwent severe volume shrinkage, and the color turned pale yellow, which was close to the color of alginate powder. The obtained composite aerogel had strong water absorption, and the volume expansion was significant after absorbing water.

### 2.3. Morphology of the Aerogel Beads

**Figure 5:**
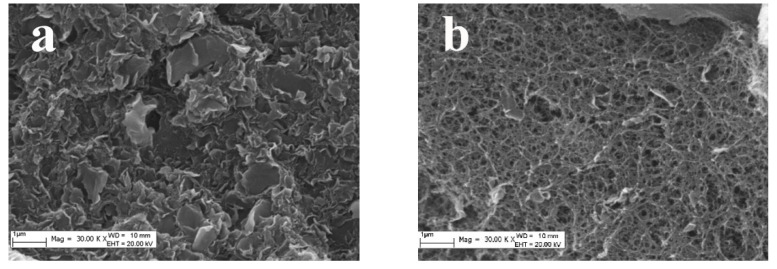
SEM images of the (**a**) xerogel and (**b**) aerogel.

Figure 5 presents the SEM images of a dry gel and a composite aerogel. It can be seen that the internal structure of the dry gel was tight; sodium alginate and bentonite were stacked together, and the edges were curled. In Figure 5b, it can be seen that the fibrous sodium alginate with a diameter of about 15 nm was connected to form an open network structure, and the pore size of the network range was 40–600 nm, wherein the pore size of about 250 nm was more distributed. After the sodium alginate solution was added to the CaCl_2_ solution dropwise, a calcium ion exchange with sodium ions occurred, resulting in the alginate chains physically crosslinking with the calcium ions and solidifying to form reinforced hydrogel network. Subsequently, the alcohol gel is obtained after the displacement of the gradient with the alcohol–water system. The alcohol gel underwent slow degassing after switching to ethanol with scCO_2_. The alcogel was replaced by scCO_2_ and slowly vented to obtain an aerogel. Due to the unique properties of scCO_2_, there was no surface tension under critical conditions, the carbon dioxide was discharged from the gel during the degassing process, and no material volume shrinkage occurred, so the network structure of the aerogel was well maintained without collapse. This result is consistent with the density data. In addition, a white flake-like substance was embedded in the matrix network of the composite aerogel and was very similar to the shape of the raw bentonite, which may be imply that it was an exfoliated platelet of bentonite.

**Figure 6:**
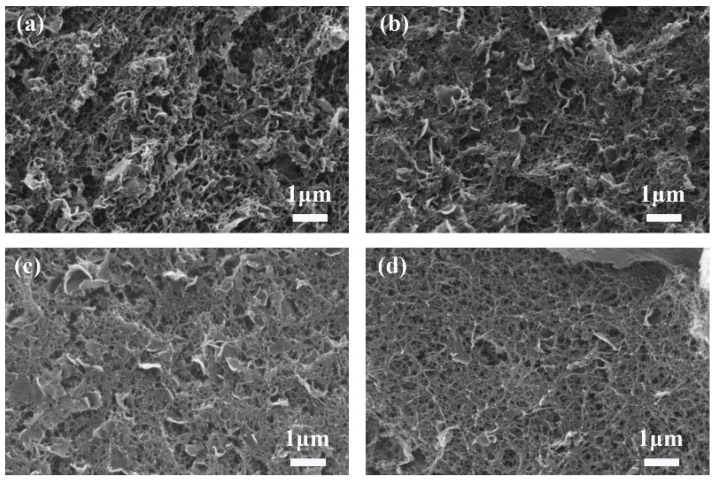
SEM images of (**a**) 1Alg-30Bt, (**b**) 2Alg-30Bt, (**c**) 3Alg-30Bt, and (**d**) 1Alg-10Bt.

Figure 6 shows an SEM image of the aerogels formed with different proportions of sodium alginate and bentonite. As seen in Figure 6, when the concentration of sodium alginate was constant at 1%, the exfoliated bentonite increased in the aerogel with the increase in bentonite content from 10% to 30%, and its dispersion was evenly distributed in the network structure of the aerogels, increasing their surface roughness. In addition, half of the bentonite flake structure existed in pores, and half was connected with sodium alginate through physical crosslinking. This was due to the ion exchange between divalent metals in the bentonite and sodium alginate, forming a crosslinking point, which caused bentonite to be more firmly combined with sodium alginate and more evenly dispersed in the sodium alginate network. When the bentonite content was fixed at 30%, the porosity of the aerogels decreased with the increase in concentration of sodium alginate solution, and more bentonite was embedded by sodium alginate. In the 1Alg-30Bt sample, a small part of the bentonite is wrapped in the sodium alginate network structure, and the rest was exposed outside the network. However, most of the bentonite samples of 3Alg-30Bt were wrapped in the Alg network structure, and the exposed part was rarely curled. This may have been because, with the increase in sodium alginate concentration, there were more crosslinked points in the system and a denser network structure, resulting in a smaller space for the free extension of bentonite lamellar, which made it difficult to curl when fixed in the SA network structure, making the composite gel structure more stable. However, when the crosslinking density was too high, the swelling of the composite aerogel was hindered, which led to the adsorption capacity of the formed aerogel decreasing. Therefore, the concentration of sodium alginate should be tuned.

### 2.4. Swelling Properties of the Alg-Bt Composite Aerogel

Because the sodium alginate concentration and bentonite content would affect the properties of the composite aerogel, the volume shrinkage through drying, swelling degree, and density depending on the gel composition were investigated. The results of when the sodium alginate concentration was constant and the percentage of bentonite was changed in the range of 10%, 30%, and 50% are shown in Table 2. The volume shrinkage of the Alg/Bt aerogels increased with the increase in bentonite content, the swelling degree decreased, and the density first increased and then decreased. When the bentonite content was constant, with the increase in sodium alginate concentration, the volume shrinkage decreased, the swelling degree decreased, and the density decreased. This indicates that the smaller the volume shrinkage, the better the structural stability of the aerogel is in the drying process, and the more regular the pore structure is. On the other hand, when the concentration of sodium alginate increased, the swelling degree decreased, and the swelling ratio of 3% alginate dropped by almost half compared to that at 1%. This may be ascribed to the increase in the crosslinking points of the gel, and the network structure was denser, resulting in the swelling degree decreasing as the concentration of sodium alginate increased from 1% to 3%. Furthermore, when the bentonite content increased from 10% to 50%, the swelling degree also slightly decreased, which may have been because a small amount of Ca^2 +^ in bentonite also participated in the crosslinking of sodium alginate. The greater the bentonite content is, the more Ca^2+^ there will be, the more crosslinking points form, and the more the swelling degree decreases [23].

### 2.5. Adsorption Properties of Complex Aerogels for Lysozyme

#### 2.5.1. Effects of pH

**Figure 7:**
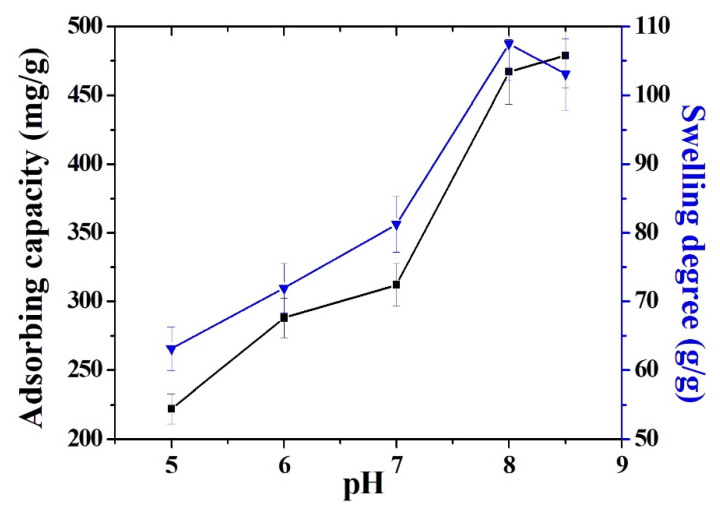
Effects of pH value on the swelling degree and the amount of lysozyme adsorbed on the composite aerogel.

In Figure 7, the adsorption capacity for lysozyme and the swelling degree in the composite aerogel are shown with respect to pH. It can be seen that the swelling had a great influence on the adsorption capacity. When the pH value was 8.5, the change tendencies of the swelling degree and the adsorption capacity were different from those of other points, probably because the lysozyme was an alkaline enzyme, and its isoelectric point was 11.0–11.35. The pH value of the solution was close to the isoelectric point of lysozyme, and the adsorption capacity was enhanced. Considering the swelling degree and adsorption capacity, the pH value of the selected phosphate buffer solution was 8.

In general, the adsorption capacity for lysozyme and the swelling degree of the composite aerogel increased with the increase in pH values from 5 to 9. In addition, it can be seen that swelling had a great influence on the adsorption capacity for lysozyme. The degree of swelling and adsorption at pH 8.5 was different from the trend at the other points, possibly because lysozyme is an alkaline enzyme, and its isoelectric point is 11.0–11.35. Alkaline enhancement brought the pH of the solution closer to the isoelectric point of lysozyme, resulting in an increase in its adsorption amount. Considering the degree of swelling and the amount of adsorption, the pH of the phosphoric acid buffer solution selected in the subsequent adsorption test was 8.

#### 2.5.2. Effects of Contact Time

**Figure 8:**
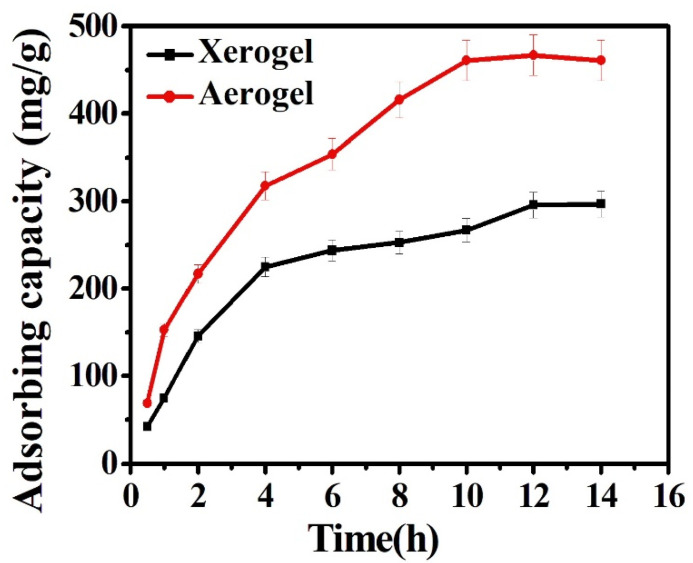
Effects of contact time on the amount of lysozyme adsorbed on the composite aerogel.

The adsorption capacities for lysozyme over time were compared by using two kinds of gels, and the results are shown in Figure 8. It was found that the aerogel reached an adsorption equilibrium of 461 mg/g at 10 h, while that of xerogel reached 268 mg/g at 12 h. The adsorption capacity of aerogel in the first 4 h was similar to that of the xerogel, and the adsorption rate was faster. Their adsorption capacities were 318 and 198 mg/g, respectively. After 4 h, the adsorption rate of the aerogels decreased, but the growth rate was still higher. The adsorptive growth rate of the xerogel decreased obviously after 4 h. This may have been due to the hierarchically porous structure of the aerogel, and the different pore structures played a critical role in adsorption with time.

#### 2.5.3. Effect of Bentonite Content

**Figure 9:**
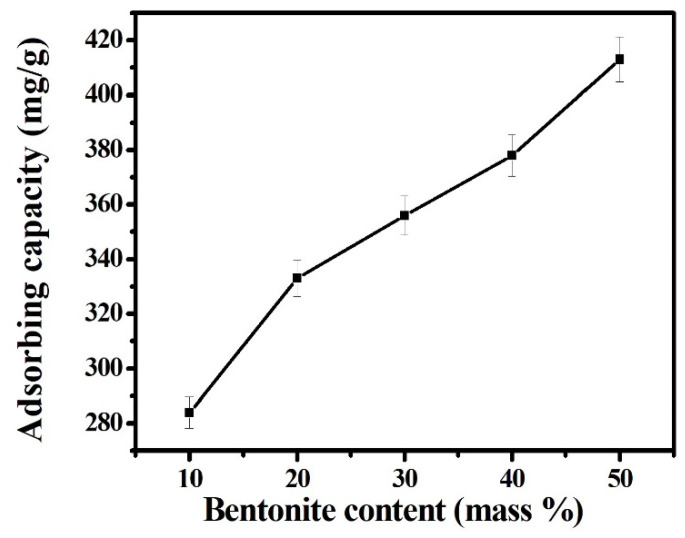
Effect of bentonite content on lysozyme adsorption.

Figure 9 shows the effect of the Bt content in the aerogel on the adsorption capacity for lysozyme. When the concentration of sodium alginate was too high, the viscosity of the sol was high, which would make the dripping of the gel beads difficult to achieve with a regular shape. In fact, when the sodium alginate solution concentration was 1% and the bentonite content reached 50%, the sol could still be dripped into regular spherical beads surrounded in CaCl_2_ solution. As seen in Figure 9, the adsorption amount was 284 mg/g when the soil content was 10%, and was further enhanced when the bentonite content reached 50%, as the adsorption amount could arrive at 413 mg/g. Obviously, with the increase in bentonite content, the adsorption amount had an almost linear relationship with bentonite content, indicating that the bentonite content in the composite aerogel had a critical impact on the adsorption of lysozyme. The swelling degree of the composite aerogel with 50% soil content and with a 1% sodium alginate concentration could reach 110 g/g. Bentonite in the form of silicate platelets has strong water absorption properties and can achieve 20–30 times its weight; its adsorption mechanisms include physical adsorption, chemical adsorption, and ion exchange; therefore, its lysozyme adsorption capacity is relatively strong. We also investigated the effects of changes in sodium alginate concentration on the swelling and adsorption properties. When the bentonite content was 30% and sodium alginate solution concentrations were 1%, 2%, and 3%, the adsorption amounts were 356, 461, and 482 mg/g, respectively. With the increase in sodium alginate concentration, the carboxyl group contained in the polymer chain increased, causing its adsorption to increase, but the trend of increase slowed down. This may have been because as the concentration of sodium alginate increased and the swelling of the gel decreased, thus slowing this growth trend.

#### 2.5.4. Effect of Initial Lysozyme Concentration on Adsorption

**Figure 10:**
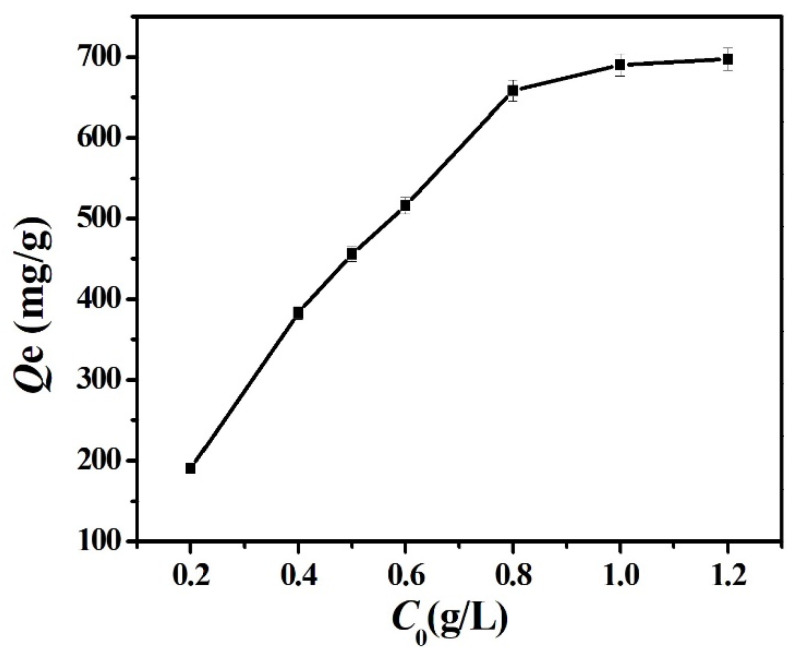
Effect of initial lysozyme concentration on adsorption capacity.

The adsorption of lysozyme under different concentrations of lysozyme solutions was evaluated. The detailed adsorption conditions were adsorbent 2Alg-30Bt, adsorption time of 10 h, and a temperature of 30 °C. The results are shown in Figure 10. When the concentration range was 0.2–0.8 g/L, the adsorption amount changed linearly with the initial concentration, from 190 to 658 mg/g. When the lysozyme solution’s concentration was higher than 0.8 g/L, the adsorption amount was close to saturation, and the equilibrium adsorption amount reached 690 mg/g. The results indicate that the Alg-Bt composite aerogel had a higher affinity to Lys than those of other reported modified clays, which may be due to their distinct textural properties and the presence of more adsorptive activity sites within the matrix [24,25].

#### 2.5.5. Adsorption Isotherm

**Figure 11:**
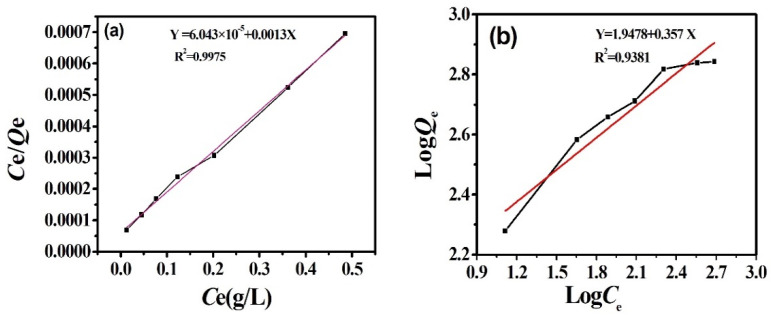
The Langmuir and Freundlich adsorption isotherm curves of lysozyme on aerogel. (**a**) Langmuir adsorption isotherm curve and (**b**) Freundlich adsorption isotherm curve of lysozyme on aerogel.

Furthermore, the above adsorption results were simulated with the Langmuir and Freundlich isothermal adsorption models, and the results are listed in Figure 11. According to the Langmuir isothermal adsorption curve, the ideal maximum adsorption capacity (*Q*_max_) was 769 mg/g, which was close to 690 mg/g at the initial concentration of lysozyme of 1.2 g/L, and the empirical parameter b of the Langmuir equation was 16.98, indicating that the adsorption capacity of the composite aerogel was very strong. According to the Freundlich isothermal adsorption curve and the fitting with the Freundlich equation, the Freundlich bond energy parameter (*K_f_*) was 88.51, and the equation’s empirical parameter n was 2.8 (0.1 < 1/n < 0.5), indicating that it was easy for the composite aerogel to adsorb lysozyme. The linear correlation coefficients R^2^ fitted with the Langmuir and Freundlich isothermal adsorption models were 0.997 and 0.938, respectively, indicating that the adsorption of lysozyme by the obtained aerogels was more in line with the Langmuir isotherm model and was a single-molecular-layer adsorption.

## 3. Conclusions

In summary, sodium alginate/bentonite composite aerogel was successfully prepared with a sol-gel and supercritical fluid drying method. This allowed volumetric shrinkage of the gel during drying to be effectively avoided and the original three-dimensional network structure to be maintained. The resulting aerogel had a large specific surface area (445 m^2^/g), a low density (0.059 g/cm^3^), and a large pore volume (3.617 cm^3^/g). When the pH of the solution was 8, the adsorption amount of lysozyme increased with the increase in bentonite content; when the initial concentration of lysozyme was 0.2–1.2 g/L, the adsorption amount first increased and then stabilized, and the equilibrium adsorption amount was as high as 690 mg/g and belonged to Langmuir isothermal adsorption. Such widely sourced biocompatible composite aerogels will have potential applications in the field of protein separation and purification.

## 4. Experimental

### 4.1. Materials and Methods

Sodium alginate (99% purity) was purchased from Acros Organics. CO_2_ with a purity of 99.9% was supplied by Wurumqi Gas Factory. Bentonite and lysozyme were purchased from Macklin Chemical Co. (Shanghai, China). CaCl_2_ of analytical reagent grade was supplied by Tianjin Chemical Reagent Co., Ltd. (Tianjin, China).

Drying of alcohol gels was carried out in a 100 mL stainless steel reactor (model FYX-0.1; Dalian Tong Chan High-Pressure Reactor Manufacturing Co., Ltd., Dalian, China). The pressure in the reactor was measured in the range of 0–30 MPa using a pressure gauge. The temperature of the reactor was controlled by a thermostat water bath (DF-101S; Jintan Medical Instruments Factory, Changzhou, China).

### 4.2. Prepared Sodium Alginate Composite Aerogels

**Scheme 1:**
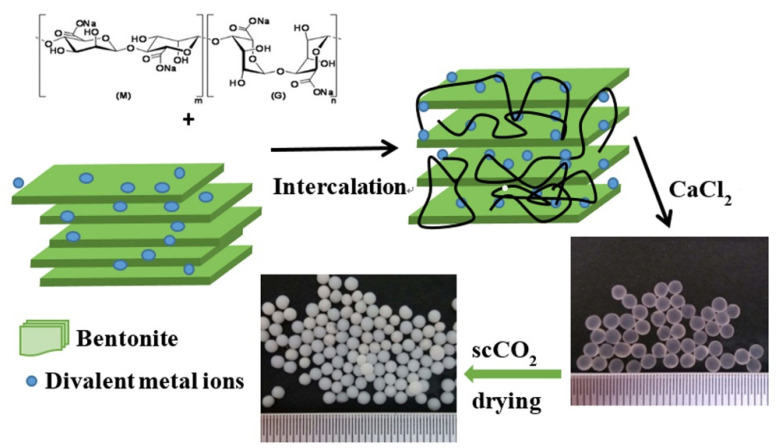
A schematic of the fabrication process on the composite aerogels.

The preparation process is shown in Scheme 1. A total of 30 mL of distilled water was taken in a 100 mL beaker, a certain amount of bentonite was added, and this was magnetically stirred for 6 h. Then, a certain amount of sodium alginate was added, which was stirred for 12 h to make a uniform mixture and allowed to stand for a period of time to remove bubbles. The above mixture was dripped into CaCl_2_ solution at 0.24 M with a syringe with a 0.8 mm needle, and it solidified for 1 h to form a gel. The gel particles were then successively put into 20%, 40%, 60%, 80%, and 100% concentrations of ethanol aqueous solution for 30 min. This resulted in an alcogel. The alcogel beads were placed into a stainless steel net and suspended in a high-pressure reactor, and the reactor was then sealed and placed in an ice-water bath, which was connected to a CO_2_ gas cylinder and filled with 90 g of liquid CO_2_, followed by an ice bath for 5 h; then, it was transferred to a 40 °C water bath for 2 h, and a sodium alginate–bentonite composite aerogel was obtained after degassing at a slow and constant speed at a constant temperature of 40 °C. Samples are identified as xAlg-yBt, where Alg denotes alginate, the value of x refers to its percentage, Bt denotes bentonite, and the value of y refers to its content.

### 4.3. Characterization

Fourier transform infrared spectroscopy (FTIR) spectra were recorded using a Vertex 70 FTIR spectrophotometer (Bruker Optics, Ettlingen, Germany). X-ray diffraction (XRD) with a Bruker D8 was used to characterize the composite aerogel with Cu-K_α_ radiation (1.5406 Å) at a generator voltage of 40 kV. SEM images were obtained using an LEO1430VP Scanning Electron Microscope (LEO, Oberkochen, Germany). The composite aerogel was embedded in an epoxy resin, and after it was cured, ultra-thin sections were performed under a microscope and then glued to a copper mesh to observe the sample slices with a Hitachi H-600 transmission electron microscope (TEM).The Brunauer–Emmett–Teller (BET) method was employed, and surface areas, pore volumes, and pore diameters of the composite aerogels were determined by using the nitrogen adsorption and desorption modes with a commercial instrument (JW-BK, Jing Wei Gao Bo Co., Ltd., Beijing, China).

### 4.4. Swelling and Density Measurements

The gel beads were first dried in vacuum at 50 °C for 12 h to remove moisture from the samples. A certain sample was accurately weighed in an Erlenmeyer flask filled with 25 mL of phosphate buffer solution. After swelling for 72 h, it was wiped with filter paper to remove excess water on the surface; then, the mass of the swollen particles was weighed, each sample was measured in parallel 3 times, and the averages were calculated. The swelling degree (*E_A_*) was calculated with the following formula:

(1) $$E_{A}=(W_{s}-W_{d})/W_{d}\times 100$$

*W_d_* is the mass of the sample before swelling and *W_s_* is the mass of the sample after swelling.

The density (*ρ*) of the aerogel was calculated as follows [26]:

*ρ* = *W_d_* / *V* (2)

where *W_d_* (g) is the mass of aerogels and *V* is the volume of aerogels. The volume was calculated with the diameter of the spherical aerogels. All measurements were in triplicate, and the error was less than 5%.

### 4.5. Lysozyme Adsorption

Lysozyme (Lys) adsorption was performed by immersing the composites in phosphate-buffered saline (PBS) containing a quantity of Lys; they were placed in Erlenmeyer flasks and incubated at room temperature for a predetermined amount of time. The Lys concentration in the solution was quantified with a predetermined standard concentration–intensity calibration curve through ultraviolet–visible spectrophotometry (UV 2550, Shimadzu Instruments Co., Ltd., Tokyo, Japan) at 280 nm. The amount of adsorbed Lys was determined through the following equation:

(3) $$Q_{e}=\frac{(C_{0}-C_{e})V}{m}$$

where *Q*_e_ is the amount of protein adsorbed (mg/g), *C*_0_ and *C_e_* are the concentrations of the Lys in the solution before and after adsorption, respectively (g/L), *V* is the volume of the protein solution (mL), and *m* is the weight of the samples (g).

In the following, the isothermal adsorption of Lys was analyzed with both the Langmuir (Equation (4)) and Freundlich (Equation (5)) equations:

(4) $$Q_{e}=\frac{Q_{\max}bC_{e}}{\left(1+bC_{e}\right)}$$

(5) $$\log Q_{e}=\log K_{f}+(1/n)\log C_{e}$$

where *Q*_max_ is the saturation adsorption capacity, *b* is an empirical parameter, *K_f_* is the binding energy constant, and n is the Freundlich constant.

## Figures and Tables

**Table 1:** Properties of the xerogel and aerogel.

Sample	Surface Area (m^2^/g)	Pore Volume (cm^3^/g)	Pore Diameter (nm)	Swelling Degree (%)	Density (g/cm^3^)
xerogel	228	0.545	9.58	78.8	1.01
aerogel	445	3.617	32.62	107.5	0.059

**Table 2:** Properties of aerogels with different ratios.

Sample	Volume Shrinkage (%)	Swelling Degree (g/g)	Density (g/cm^3^)
1Alg-10Bt	19	135.5	0.059
1Alg-30Bt	46	118.5	0.363
1Alg-50Bt	53	111	0.227
2Alg-30Bt	44	107.5	0.282
3Alg-30Bt	17	61.2	0.221

## Data Availability

Data sharing is not applicable to this article.
